# Ligature of the Brachial Artery for the Aneurism from Blood-Letting

**Published:** 1835

**Authors:** 


II. *Ligature of the Brachial Artery for the Aneurism from Bloodletting.*—In the highly respectable periodical just quoted, we have a case, reported by the indefatigable professor Smith, of the University of Maryland, in which he resorted to this operation with success. Pressure above and upon the tumor, although it arrested the pulsation and emptied the sac did not effect a cure.

"The operation was performed in the presence of the medical class of the University of Maryland, and with the assistance of the pupils of the house. The patient was seated in a chair, and his arm was extended upon a table. The incision was made as usual along the border of the biceps, the fascia of the arm being laid bare at the first stroke of the knife, and opended by the second. On introducing the finger into the wound, I felt the pulsation of two arteries—the one in the usual position of the brachial, close beneath the border of the biceps—the other toward the ulnar border of the arm, but not remote from it. On compressing the latter, the pulsations of the tumor did not cease, but when this was practised upon the former, they were instantly commanded. The pulsations of these vessels were so equally strong that I immediately inferred that, in this instance, the division of the brachial had occurred at a higher point than usual, and that only the radial branch was concerned in the operation. With this impression I immediately applied the ligature to the artery beneath the biceps. The pulsations of the tumor at once ceased, its volume diminished, and it became flaccid. A silk ligature was employed, one extremity of which was left hanging from the wound. The incision was very accurately closed by means of adhesive strips—a compress was applied to the tumor, and a roller to the member, from the hand to the shoulder. The patient was kept in a tranquil state. At the same hour of the next day the tumor was found, on examination, to pulsate feebly. There was also a pretty strong pulsation in the radial artery, at the wrist. The brachial artery was also found to pulsate slightly where it merged itself in the tumor. I could not now feel the pulsation of an artery toward the ulnar border of the arm, which could be regarded as the ulnar artery, nor were the pulsations of the ulnar at the wrist stronger than those of the radial, and I now came to the conclusion that the collateral vessel, which I so distinctly felt during the operation, was the anastomotic, considerably enlarged in consequence of the circulation in the brachial. There had occurred no visible diminution of temperature in the limb. The bandage was re-applied and kept wet with cold water.

On the third day the bandage was again removed, and we were gratified to discover that there now no longer existed any pulsation in the tumor, although it was still manifest in the artery both above and below it.

On the fifth day there was still no pulsation in the tumor, and it had now become quite hard and incompressible. There was not much tenderness to the touch, nor had the patient experienced much pain, though it was occasionally such as to disturb his rest at night.

On the thirteenth day the ligature came away. In the mean time complete reunion of the wound by adhesion had taken place, and he had scarcely felt a sensation where the ligature had been applied, till the present time. By any prudent effort we could not now, by pressure, force any fluid out of the cyst into the artery, or in any measure diminish its size. It was manifest, therefore, that the blood contained in the cyst had now formed a firm coagulum, and that we might confidently expect its ultimate obliteration.

On the twentieth day the patient complained of severe pain and tenderness in the tumor, and evidently a slight degree of inflammatory swelling had taken place at its base. I conjectured that suppuration of the cyst might be about to happen, in consequence of the coagulum being so thickly covered with integuments. I directed a poultice to be applied to the part, and this to be compressed with the bandange. In two or three days the inflammation was wholly dissipated, and I found on comparing the tumor with a cast which had been made of the part before the operation, that it had lost half its original size. It was perfectly free from pulsation."

